# The lower incidence of endometrial cancer after sodium-glucose cotransporter 2 inhibitors administration in type 2 diabetes mellitus population: a nationwide cohort study

**DOI:** 10.7150/ijms.95584

**Published:** 2024-05-19

**Authors:** Po-Jen Yang, Po-Hui Wang, Jing-Yang Huang, Chia-Yi Lee, Chiao-Wen Lin, Chung-Yuan Lee, Shun-Fa Yang

**Affiliations:** 1 School of Medicine, Chung Shan Medical University, Taichung, Taiwan.; 2 Department of Family and Community Medicine, Chung Shan Medical University Hospital, Taichung, Taiwan.; 3 Institute of Medicine, Chung Shan Medical University, Taichung, Taiwan.; 4 Department of Obstetrics and Gynecology, Chung Shan Medical University Hospital, Taichung, Taiwan.; 5 Department of Medical Research, Chung Shan Medical University Hospital, Taichung, Taiwan.; 6 Department of Ophthalmology, Nobel Eye Institute, Taipei, Taiwan.; 7 Institute of Oral Sciences, Chung Shan Medical University, Taichung, Taiwan.; 8 Department of Obstetrics and Gynecology, Chiayi Chang Gung Memorial Hospital Chiayi, Taiwan.; 9 Department of Nursing, Chang Gung University of Science and Technology, Chiayi Campus, Chiayi, Taiwan.

**Keywords:** SGLT2 inhibitors, endometrial cancer, interval, type 2 diabetes mellitus, oxidative stress

## Abstract

The Sodium-glucose co-transporter 2 (SGLT2) inhibitor is an anti-glycemic agent that frequently used in type 2 diabetes mellitus (T2DM) with antioxidant effects. Endometrial cancer (EC) is a common gynecological malignancy that correlates with oxidative stress. The aim in the present study is to survey the potential association between the SGLT2 inhibitor administration and the incidence of EC by the application of the National Health Insurance Research Database (NHIRD) of Taiwan. A retrospective cohort study was directed and the T2DM participants were divided into the SGLT2 inhibitors users and non-SGLT2 inhibitors users. After matching, a total of 163,668 and 327,336 participants were included into the SGLT2 inhibitors and control groups, respectively. The primary outcome is regarded as the development of EC according to the diagnostic, image, and procedure codes. Cox proportional hazard regression was employed to generate the adjusted hazard ratio (aHR) and 95% confidence interval (CI) of EC between the two groups. There were 422 and 876 EC events observed in the SGLT2 inhibitors and control groups, respectively. The SGLT2 inhibitors group demonstrated a significantly lower incidence of EC formation compared to the control groups (aHR: 0.87, 95% CI: 0.76-0.99). In the subgroup analysis, the correlation between SGLT2 inhibitor administration and lower rate of EC existed in the T2DM individuals with aged under 60. Moreover, the association between SGLT2 inhibitor administration and lower EC incidence only presented in the T2DM population with SGLT2 inhibitor administration under one year (aHR: 0.58, 95% CI: 0.45-0.73). In conclusion, the administration of SGLT2 inhibitors correlates to lower incidence of EC in T2DM population.

## Introduction

Type 2 diabetes mellitus (T2DM) is a metabolic disease that features with blood glucose increment and affects a large proportion of people 1. The endogenous resistance of human body cells to insulin is the main mechanism for the persistent hyperglycemia and subsequent T2DM development 2. For the therapy of T2DM, oral medications like the biguanides and alpha-glucosidase inhibitors can be used, while insulin injection would be administrated for individuals with advanced T2DM 3. Sodium-glucose cotransporter 2 (SGLT2) inhibitors had been frequently administered for T2DM treatment recently with glycolated hemoglobin retardation from 0.5 to 1.0 percent 4, 5.

Except for anti-hyperglycemic ability, SGLT2 inhibitors can prevent other morbidities based on earlier literatures 4, 6. Kidney function concerning glomerular filtration rate can benefit from the administration of SGLT2 inhibitors 7. The possibility of myocardial infarction would be decreased in the T2DM populations taking SGLT2 inhibitors as anti-glycemic therapy 8. Moreover, the application of SGLT2 inhibitors correlates to neuroprotective consequences which may be considered for cognitive impairment therapy 9. In the field of ophthalmology, administration of SGLT2 inhibitors is associated with few episodes of dry eye disease and diabetic retinopathy formations 10-13.

Endometrial cancer (EC) is derived from the inner uterine epithelium and is the most prevalent gynecological cancers in developed countries with trend of increment 14-16. The known risk factors for EC include old age, smoking, obesity, previous usage of hormone replacement therapy and endometrial hyperplasia 17-19. Nevertheless, there was rarely evidence for the association between SGLT2 inhibitor administration and the incidence of EC. Since SGLT2 inhibitor administration can reduce the oxidative stress which is associated with the existence of EC 20, 21, a potential association between them may present.

Consequently, the purpose of present study is to evaluate the possible correlation between the SGLT2 inhibitor administration and following EC incidence in T2DM population via the National Health Insurance Research Database (NHIRD) of Taiwan. Subgroup analysis for T2DM populations with different age and SGLT2 inhibitors administration duration was also conducted.

## Materials and Methods

### Data source

The present study adhered to the declaration of Helsinki in 1964 and late amendments, and the present study was allowed by National Health Insurance Administration of Taiwan and the Institutional Review Board of Chung Shan Medical University Hospital (Project code: CS1-20113). The essentiality of written informed consent was waived by the institutions mentioned above. The NHIRD of Taiwan owns claimed medical documents of nearly 23 million Taiwanese during the interval from January 1, 2015 to December 31, 2021. The following items are the documents available in Taiwan NHIRD: International Classification of Diseases-Ninth Revision (ICD-9), International Classification of Diseases-Tenth Revision (ICD-10) diagnostic codes, age, sex, urbanization degree, education degree, income level, image exam codes, medical department codes, laboratory exam codes, procedure as well as surgery codes and the international ATC codes for prescribed medications.

### Participant Selection

**Figure 1:**
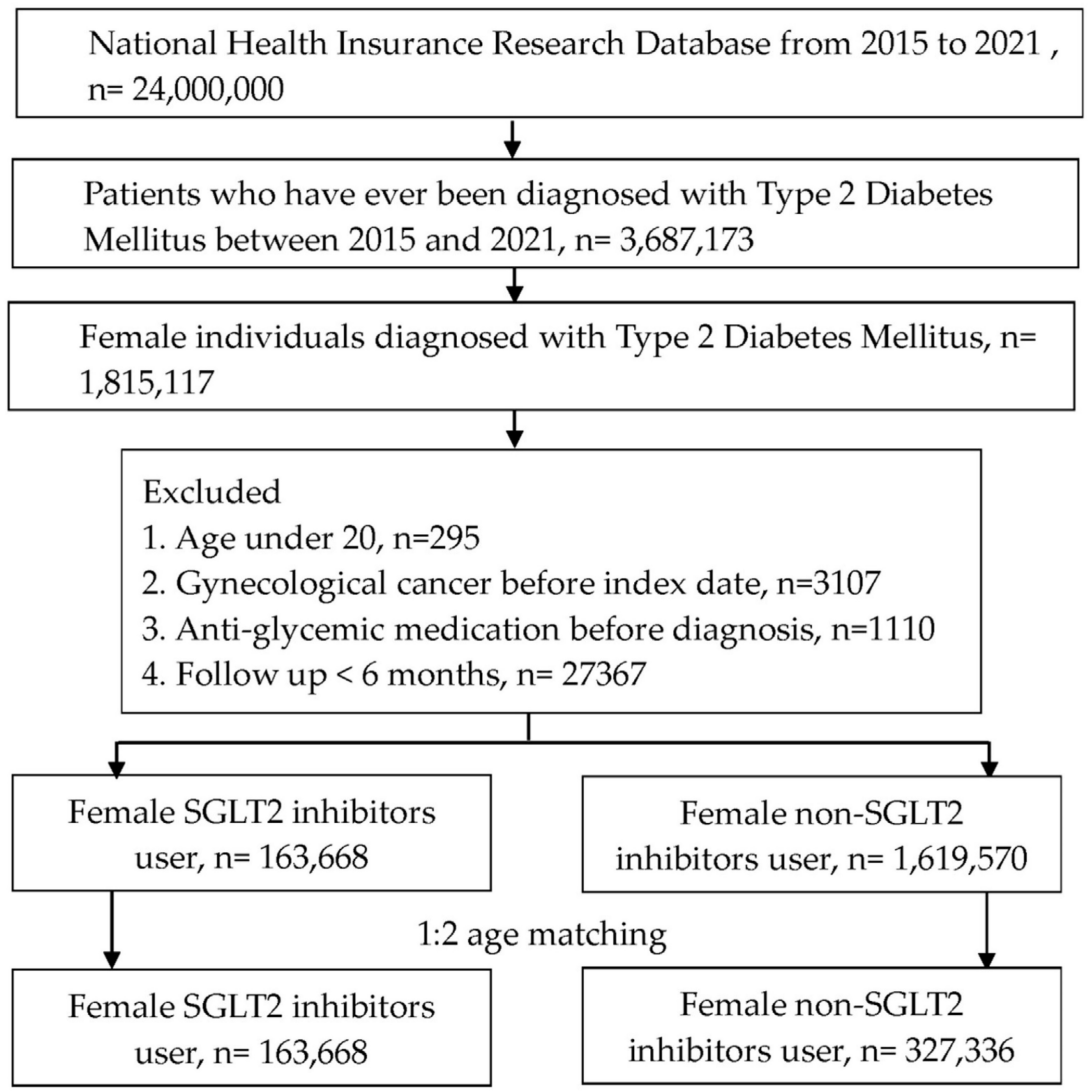
Flowchart of participant selection. NHIRD: National Health Insurance Research Database, N: number, T2DM: type 2 diabetes mellitus, SGLT2: sodium-glucose cotransporter 2.

A retrospective cohort study was organized. We defined the participants as T2DM with SGLT2 inhibitors administration by these criteria: (1) the presence of ICD-9/ICD-10 codes of T2DM diagnosis from 2015 to 2021, (2) the presence of glycated hemoglobin exam before the T2DM diagnosis, (3) visits internal physician with an interval longer than three months, and (4) administration of SGLT2 inhibitors involving canagliflozin, dapagliflozin, empagliflozin or ertugliflozin based on the ATC codes. The index date of the present study was 6 months after SGLT2 inhibitors administration. The coming exclusion criteria were used to homogenize the T2DM participants: (1) age under 20 years or over 100 years, (2) the existence of gynecological or breast cancers before the index date, (3), anti-glycemic drugs prescribed before T2DM diagnosis and (4) no demography. After that, one T2DM participant with SGLT2 inhibitors administration was matched to two T2DM individuals without SGLT2 inhibitor administration and the latter group served as the control group. Finally, a total of 163,668 and 327,336 T2DM participants were categorized into SGLT2 inhibitors group and control group, respectively. The flowchart of participant selection is revealed in Figure 1.

### Primary outcome

The primary outcome of the present study was EC formation which achieved following conditions: (1) the presence of ICD-9/ICD-10 diagnostic codes that implied EC diagnosis, (2) the arrangement of pelvic exam, transvaginal ultrasound or hysteroscopy before the EC diagnosis via image codes, (3) the arrangement of dilation and curettage procedure or endometrial biopsy before the EC diagnosis via procedure/surgery codes, and (4) the EC diagnosis was confirmed by a gynecologist. The T2DM participants in the present study were tracked until the EC diagnosis, participants withdrew from this National Health Insurance or the deadline of Taiwan NHIRD which implies December 31, 2021.

### Confounder adjustment

Multiple confounders were put in the multivariable analysis to adjust the effects of those confounders on the EC formation: age, occupation, hypertension, coronary heart disease, hyperlipidemia, ischemic stroke, hemorrhage stroke, kidney disease, rheumatoid arthritis, systemic lupus erythematosus, Sjogren syndrome, ankylosing spondylitis and chronic obstructive pulmonary disease by the ICD-9 or ICD-10 diagnostic codes. Furthermore, multiple anti-hyperglycemic medicines such as biguanides, sulfonylureas, alpha glucosidase inhibitors, statin, thiazolidinediones, dipeptidyl peptidase-4 (DPP4) inhibitor, and insulin were enrolled in the multivariable analysis by ATC codes. To assure the associated morbidities and anti-hyperglycemic medicine duration in T2DM participants are long enough to impact EC incidence, only associated morbidities and anti-hyperglycemic medicine that existed in NHIRD over two years before our index date were included.

### Statistical analysis

Descriptive analyses were employed to demonstrate the demographic data, associated morbidity and anti-hyperglycemic medicine distributions between the SGLT2 inhibitors and control groups, respectively. Absolute standardized difference (ASD) was employed to evaluate the difference of confounders between the SGLT2 inhibitors and control groups, and ASD value over 0.1 was defined as significant difference. Then Cox proportional hazard regression was used to generate the adjusted hazard ratios (aHR) as well as 95% confidence intervals (CI) of the EC formation between the two groups, and the impact of demographic data, associated morbidity and anti-hyperglycemic medicine were all adjusted in the regression model. Concerning the subgroup analyses, the T2DM participants were stratified by age (60 years) as well as SGLT2 inhibitors administration period (1 years), and Cox proportional hazard regression was employed again to generate the incidence of EC formation between SGLT2 inhibitor users and non-SGLT2 inhibitor users. Then interaction test was performed to investigate the impact of SGLT2 inhibitors on EC between different subgroups. Statistical significance was delineated as P < 0.05 in the present study.

## Results

The baseline characters of the SGLT2 inhibitors population and non-SGLT2 inhibitors population are presented in Table 1. The distributions of both age and occupation did not reveal significant difference between the SGLT2 inhibitor and control groups (both ASD < 0.1). For the associated morbidities, all the systemic and gynecological diseases illustrated similar percentages between the SGLT2 inhibitor group and control group due to the match process (all ASD < 0.1). The usage of anti-diabetic medications including biguanides, sulfonylureas, alpha glucosidase inhibitors, thiazolidinediones, DPP4 inhibitor, insulin and statin presented with a significantly higher rate in the SGLT2 inhibitors group than the control group (all ASD > 0.1) (Table 1).

There were 422 and 876 EC events developed in the SGLT2 inhibitors and control groups after the whole follow-up period, respectively. The SGLT2 inhibitors group demonstrated a significantly lower incidence of EC compared to the control group according to Cox proportional hazards regression (aHR: 0.87, 95% CI: 0.76-0.99) (Table 2). Concerning the subgroup analysis, the SGLT2 inhibitor users showed a significantly lower rate of EC formation compared to the non-SGLT2 inhibitor users in under 60 years old (Table 3). On the other side, SGLT2 inhibitor administration shorter than one year correlated to significantly lower EC incidence than non-SGLT2 inhibitors in T2DM population (aHR: 0.58, 95% CI: 0.45-0.73) (Table 3).

## Discussion

Briefly, the present research demonstrated a significantly lower incidence of EC development in the T2DM participants with SGLT2 inhibitors administration than those without SGLT2 inhibitors administration. Moreover, the association between SGLT2 inhibitors administration and EC incidence are in under 60 years old populations.

Administration of SGLT2 inhibitors is associated with the retardation of several disorders throughout the human body 7, 8, 12. The crucial function of SGLT2 inhibitor is its anti-glycemic activity which can decrease the concentration of glycated hemoglobin for approximately 1.0 percent 4. In the combined therapy of SGLT2 inhibitors and the DPP-4 inhibitor, additional decrement of 0.71 percent glycated hemoglobin concentration was found in contrast to the DPP-4 inhibitor mono-therapy 22. Except for the serum glucose regulation, administration of SGLT2 inhibitors is associated with lower degree of inflammation in the condition of autoimmune myocarditis 23. Regarding the mechanism of anti-inflammatory ability, SGLT2 inhibitors diminished the expression of adipose tissue-mediated inflammation and pro-inflammatory cytokines 6. Except for the above two mechanisms, SGLT2 inhibitor can serve as an antioxidant and diminish the reactive oxygen species production in diabetic kidney disease circumstances 21. Moreover, Monnier *et al.* suggest that glucosidase inhibitors could reduce mean amplitude of glucose excursions (MAGE), and reduce oxidative stress and free radical damage 24. For the pathophysiology of EC, the presence of high oxidative stress is related to the development of EC 20. Oxidative stress can regulate genetic expression during EC formation 25. Also, the expression of tumor necrosis factor-α and C-reactive protein was involved in the pathogenesis of EC 26, and inflammatory indexes like leukocytosis and thrombocytosis relate to the prognosis of EC 27. Furthermore, hyperglycemic status and diabetes can increase the risk of EC 28, while diabetes risk reduction diet can also reduce the risk of EC formation 29. Since SGLT2 inhibitors can defer the mechanism related to EC formation 4, 21, we speculate that the administration of SGLT2 inhibitors is associated with lower incidence of EC formation. This hypothesis was further supported by the results of the present study.

In the present study, the administration of SGLT2 inhibitors in the T2DM participants related to lower incidence of EC formation. According to the earlier publications, SGLT2 inhibitors could diminish the proliferation of several cancer cells such as cells derived from breast and liver cancers 30, 31. Still, the correlation between SGLT2 inhibitor administration and the formation of gynecological carcinoma like EC had not been investigated. To our knowledge, the results of the present study might be preliminary evidence illustrating the association between SGLT2 inhibitor administration and lower incidence of EC formation in T2DM individuals. Moreover, we erased the T2DM participants diagnosed with EC within 6 months after SGLT2 inhibitors administration, thus the time order between SGLT2 inhibitor administration and EC formation might be confirmed. In addition, a number of well-known risk factors like the age, endometrial hyperplasia and the polycystic ovarian syndrome were adjusted in the Cox proportional hazards regression of the present research 17-19. Consequently, SGLT2 inhibitor administration may be an independent defensive factor for the formation of EC. In an earlier study, the decrement of liver cancer cell proliferation under SGLT2 inhibitor administration may have resulted from reduced oxidative stress 31, which may also responsible for the lower incidence of EC in the present study.

For the epidemiological condition of T2DM, the T2DM may be the most frequent metabolic disease which disturbs over 10 percent of human population in earlier article 3. Moreover, the incidence of T2DM has gradually grown in which approximately 700 million humans may suffer from T2DM by 2040 2. SGLT2 inhibitors have demonstrated hyperglycemic regulation capabilities and have been frequently administered as T2DM therapy 3, 32. Above 10 percent of individuals with T2DM in the US are applying for SGLT2 inhibitors to decrease the severity of T2DM 33. EC is also a common gynecological disease which 142,000 women are diagnosed with annually 34, 35. The mortality of EC is about 2.0-3.7 per 100,000 women 15, and 42,000 women die of EC each year 34. Since both T2DM patients with SGLT2 inhibitor administration and EC individuals account for a large proportion of population, any relationship between these two populations might be demonstrated.

There are still several limitations in the present study. Firstly, the NHIRD of Taiwan is a claimed database without real medical documents. Accordingly, several crucial data involving the real serum glucose and glycated hemoglobin concentrations in T2DM participants, the trends of blood sugar changes in T2DM patients, the treatment responses after anti-glycemic therapy of T2DM patients, the actual sites of EC, the image results of EC, the pathological type of EC, the therapeutic outcomes of EC, the recurrence of EC, and details of other systemic and gynecological disorders could not be analyzed in the present study. Secondly, the retrospective nature of Taiwan NHIRD and our study design would reduce the homogeneity of the participants compared to a prospective one. The administration of hormone replacement therapy and oral contraceptives can alter the risk of EC development 34, but management of these is self-paid in Taiwan and not available in the NHIRD. Finally, smoking and obesity are also predisposing factors for EC formation 17, but the physicians rarely entered the related ICD-9/ICD-10 codes into the insurance system thus we also did not analyze smoking and obesity otherwise severe underestimation of the two confounders could occur.

In conclusion, the administration of SGLT2 inhibitors in T2DM population correlates to significantly lower incidence of EC after adjusting for multiple confounders. Consequently, SGLT2 inhibitors might be recommended for the T2DM patients with preexisting risk factors of EC formation. Further large-scale prospective study to analyze the association between SGLT2 inhibitors administration and treatment outcome of EC is necessary.

## Figures and Tables

**Table 1:** The characters in SGLT2 user and matched comparison

Character	Control group (N: 327336)	SGLT2 inhibitor group (N: 163668)	ASD
Age			0.0000
20-39	17670 (5.40%)	8835 (5.40%)	
40-49	36722 (11.22%)	18361 (11.22%)	
50-59	80116 (24.48%)	40058 (24.48%)	
60-69	111622 (34.10%)	55811 (34.10%)	
70-79	60504 (18.48%)	30252 (18.48%)	
>=80	20702 (6.32%)	10351 (6.32%)	
Occupation			0.0280
Government employee	10895 (3.33%)	5316 (3.25%)	
Worker	181764 (55.53%)	93260 (56.98%)	
Farmer and fisherman	55599 (16.99%)	25702 (15.70%)	
Others	79078 (24.16%)	39390 (24.07%)	
Co-morbidity			
Hypertension	169611 (51.82%)	101028 (61.73%)	0.2011*
Coronary heart disease	30924 (9.45%)	23051 (14.08%)	0.1443*
Hyperlipidemia	167061 (51.04%)	108528 (66.31%)	0.3140*
Ischemic stroke	13958 (4.26%)	7799 (4.77%)	0.0241
Hemorrhage stroke	2289 (0.70%)	1164 (0.71%)	0.0014
Kidney disease	32156 (9.82%)	16497 (10.08%)	0.0086
Rheumatoid arthritis	3557 (1.09%)	1449 (0.89%)	0.0204
Systemic lupus erythematosus	736 (0.22%)	252 (0.15%)	0.0163
Sjogren syndrome	3856 (1.18%)	1471 (0.90%)	0.0276
Ankylosing spondylitis	2489 (0.76%)	1185 (0.72%)	0.0042
chronic obstructive pulmonary disease	8539 (2.61%)	4241 (2.59%)	0.0011
Co-medication			
Biguanides	179428 (54.81%)	149456 (91.32%)	0.9028*
Sulfonylureas	73873 (22.57%)	66089 (40.38%)	0.3908*
Alpha glucosidase inhibitors	22845 (6.98%)	27730 (16.94%)	0.3107*
Thiazolidinediones	23704 (7.24%)	29103 (17.78%)	0.3227*
DPP4 inhibitor	54086 (16.52%)	62791 (38.36%)	0.5048*
Insulin	38605 (11.79%)	39236 (23.97%)	0.3219*
Statin	161792 (49.43%)	119710 (73.14%)	0.5020*

ASD: absolute standard difference, DPP4: dipeptidyl peptidase-4, N: number, SGLT2: sodium-glucose cotransporter 2 * Denotes significant difference between groups

**Table 2:** The risk of endometrial cancer between SGLT2 inhibitor and non-SGLT2 inhibitor users

Endometrial cancer event	Control group	SGLT2 inhibitor group
Person-months	11182107	5702019
Event	876	422
Crude HR (95% CI)	Reference	0.95 (0.84-1.06)
aHR (95% CI)	Reference	0.87 (0.76-0.99)

aHR: adjusted hazard ratio, CI: confidence interval, SGLT2: sodium-glucose cotransporter 2

**Table 3:** Subgroup analysis divided by age and duration of SGLT2 inhibitors administration

Subgroup	aHR	95% CI	Interaction P
Age			0.7954
< 60 years	0.69	0.57-0.82	
≧ 60 years	1.17	0.94-1.37	
SGLT2 inhibitor duration			<0.0001
< 1 years	0.58	0.45-0.73	
≧ 1 years	1.03	0.88-1.20	

aHR: adjusted hazard ratio, CI: confidence interval, SGLT2: sodium-glucose cotransporter 2
